# Improved outcomes for hepatic trauma in England and Wales over a decade of trauma and hepatobiliary surgery centralisation

**DOI:** 10.1007/s00068-017-0765-y

**Published:** 2017-02-16

**Authors:** J. Barrie, S. Jamdar, M. F. Iniguez, O. Bouamra, T. Jenks, F. Lecky, D. A. O’Reilly

**Affiliations:** 1 Department of Hepato-pancreatobiliary Surgery, Manchester Royal Infirmary, Central Manchester Foundation Trust, Oxford Rd, Manchester, M13 9WL UK; 2 Trauma Audit and Research Network (TARN), Manchester Academic Health Science Centre, The University of Manchester, Manchester, M6 8HD UK; 3 EMRiS Group, HSR Section, School of Health and Related Research, University of Sheffield, Sheffield, UK; 4 School of Medical Sciences, The University of Manchester, Manchester, UK

**Keywords:** Liver, Trauma, Outcomes, Hepatobiliary, Centralisation

## Abstract

**Background:**

Over the last decade trauma services have undergone a reconfiguration in England and Wales. The objective is to describe the epidemiology, management and outcomes for liver trauma over this period and examine factors predicting survival.

**Methods:**

Patients sustaining hepatic trauma were identified using the Trauma Audit and Research Network database. Demographics, management and outcomes were assessed between January 2005 and December 2014 and analysed over five, 2-year study periods. Independent predictor variables for the outcome of liver trauma were analysed using multiple logistic regression.

**Results:**

4368 Patients sustained hepatic trauma (with known outcome) between January 2005 and December 2014. Median age was 34 years (interquartile range 23–49). 81% were due to blunt and 19% to penetrating trauma. Road traffic collisions were the main mechanism of injury (58.2%). 241 patients (5.5%) underwent liver-specific surgery. The overall 30-day mortality rate was 16.4%. Improvements were seen in early consultant input, frequency and timing of computed tomography (CT) scanning, use of tranexamic acid and 30-day mortality over the five time periods. Being treated in a unit with an on-site HPB service increased the odds of survival (odds ratio 3.5, 95% confidence intervals 2.7–4.5).

**Conclusions:**

Our study has shown that being treated in a unit with an on-site HPB service increased the odds of survival. Further evaluation of the benefits of trauma and HPB surgery centralisation is warranted.

## Introduction

In 2010, there were 5.1 million deaths from injuries (almost 1 out of every 10 deaths in the world) [1]. In the United Kingdom trauma remains a leading cause of death and disability, particularly among young adults [2]. There have been significant changes in the approach to the management of liver trauma over time [3]. The period of aggressive surgical treatment that arose from the second world war changed to one of conservative non-operative management including angio-embolisation and, when laparotomy was performed, damage control strategies including perihepatic packing from the mid 1980s onwards [3]. Nonetheless, liver injury remains the main cause of death in patients with severe abdominal trauma [4] and mortality rates from contemporary series of hepatic trauma range from 5 to 42% depending on inclusion criteria [3, 5–12]. The major cause of death in patients with hepatic injury remains uncontrolled haemorrhage from the liver or from concomitant injuries [10, 13].

Alongside advances in the management strategies for injured patients has been the realisation that regional trauma systems and centralised trauma units save lives, from literature from the USA [14, 15] and Australia [16]. The UK has lagged behind, with the 2007 National Confidential Enquiry into Patient Outcome and Death **(**NCEPOD) study “Trauma: Who Cares?” reporting that more than half the 795 patients examined were subjected to less than good practice [17]. There was greater room for improvement with organisational rather than clinical aspects of care. This gave added impetus to the drive for trauma systems and trauma centres. The revised National Health Service Operating Framework for 2011–2012 stated that all regions should move towards networks for trauma. Currently there are 32 registered Major Trauma Centres (MTCs) in the UK, four centres went live in London in 2010 and in the rest of the UK from April 2012 (with different live dates for each) [18].

The objective of this study is to describe the current demographic profile, epidemiology and management of hepatic trauma in England and Wales in a contemporary series from 2005 to 2014, a decade spanning reconfiguration of trauma services.

## Methods

### Data collection

The Trauma Audit and Research Network (TARN) database was interrogated for data on patients coded as hepatic trauma. Hepatic trauma patients were defined as those sustaining any of the Abbreviated injury scale (AIS) codes 5418: 10, 12, 14, 20, 22, 24, 26, 28, 30, 40 and 99. TARN is a collaboration of hospitals from England, Wales and The Republic of Ireland, with some membership in continental Europe. TARN is the largest trauma registry in Europe, with more than 500,000 separate patient records. TARN aims to collect and analyse clinical and epidemiological data and thereby provide a statistical base to support clinical audit, to aid the development of trauma services and to inform the research agenda. TARN began in 1989 and has grown from an initial group of 13 hospitals to 100% of trauma receiving hospitals across England and Wales.

Patient inclusion was based on all those patients over 16 years of age coded as hepatic trauma who either: had a length of stay greater than 3 days, were admitted to an intensive care area, died or were transferred for specialist care. Patients with known outcome only were included. TARN excludes all simple isolated limb injuries and isolated fractured neck of femur/pubic ramus in patients aged >65 years. Patients meeting the above criteria were initially identified in the accident and emergency department and followed up by a local TARN co-ordinator who collected details on patient demographics, type and mechanism of injury, haemodynamic stability on presentation, injuries sustained, management and outcome. Injuries were described and scored using the Abbreviated Injury Scale (AIS) 2005 revision described by the Association for the Advancement of Automotive Medicine.

Patients who presented over a 10-year period were studied (January 2005–December 2014) in terms of demographics, mechanism of injury, clinical status on arrival and management. Outcome at 30 days was analysed. Five chronological 2 year periods were examined in detail; 2005–2006, 2007–2008, 2009–2010, 2011–2012 and 2013–2014.

### Data validation and quality assurance

Data validation and quality assurance was achieved as previously described [19]. Briefly, an online electronic data collection and reporting system (EDCR) has been in use since September 2005. The EDCR prevented users from dispatching submissions with incomplete obligatory data fields. Validation procedures are in place to check for accuracy in date/time sequencing, physiological measurements and investigations. All coders are trained in injury coding and have their work subjected to validation and internal quality checks, on a weekly basis.

### Statistical analysis

Differences between patient subgroups were tested by using the Chi square test, Fisher’s exact test and Mann–Whitney tests. Two-tailed *P* values of less than 0.05 were considered statistically significant. All analyses were undertaken using SPSS software, version 16. Independent predictor variables for the outcome of liver trauma were analysed using multiple logistic regression. Overall model fit assessment was with the Hosmer and Lemeshow (goodness of fit) test.

## Results

### Frequency and clinical features of liver injury

Of 211,934 total records in the TARN database between January 2005 and December 2014, 4697 patients sustained hepatic trauma with 4368 (93%) having a known, recorded outcome. Results are based on the 4368 patients with a known outcome. Median age was 34 years (interquartile range 23–49). Gender distribution remained unchanged during this period, with 3094 (71%) patients being male. In each year of the study over 50% of injuries were due to road traffic collisions. The mechanism of injury for the overall series is shown in Table 1. Median liver injury severity score by the American Association for the Surgery of Trauma (AAST) grade was II (IQR II–III). 903 (20.7%) cases were isolated liver injuries.

**Table 1:** Demographics, management and outcomes of isolated liver injury compared to polytrauma from 2005 to 2014

	Polytrauma	Isolated liver injury
Age	33.5 (23.4–48.8)	33.8 (23.6–48.8)
Male	2505 (72.3%)	589 (65.2%)
Female	960 (27.7%)	314 (34.8%)
Blunt	2961 (85.5%)	582 (64.5%)
Penetrating	504 (14.5%)	321 (35.5%)
Liver Surgery	193 (5.6%)	48 (5.3%)
ISS	29 (19–41)	8 (4–12)
Alive	2771 (80%)	882 (97.7%)
Dead	694 (20%)	21 (2.3%)

### Management and outcomes

3581 (82%) patients underwent computed tomography (CT) scanning as part of their work-up. Median Time to CT was 1 h (IQR 0.48–2.27 h). 2861 (65.5%) of patients were reviewed by a consultant in the emergency department. Tranexamic acid was given in the emergency department in 21.3% of cases (932 patients). 56% of patients underwent some kind of operative intervention but only 5.5% of patients (*n* = 241) required a liver specific operation, including liver packing, a repair of liver laceration or liver resection. 60% of patients were admitted to critical care (*n* = 2619) with a median length of critical care stay of 4 days (IQR 2–10 days). Median length of hospital stay was 9 days (IQR 5–20). Demographics, management and outcomes for isolated liver injuries as compared to those injuries sustained as part of a polytrauma are shown in Table 1. Isolated injuries had better survival rates despite a higher proportion of penetrating injuries. 715 patients died over the decade, with the overall 30-day survival rate being 83.6%.

### Management and outcomes based on time periods

Five chronological 2-year periods were examined in detail; 2005–2006, 2007–2008, 2009–2010, 2011–2012 and 2013–2014. Clinical features and management are shown for each period in Table 2. Age, gender, mechanism of injury and clinical features such as injury severity, remained relatively stable throughout each study period. Chronological improvements in management were found in terms of consultant review in the emergency department, use of tranexamic acid and frequency and promptness of CT scanning. There appears to be an increase in patients undergoing liver specific surgery over the five time periods. The demographics, management and outcomes for these patients is shown in Tables 3 and 4. 84.8% of patients were alive at 30-days in 2013–2014 compared to 75.3% in 2005–2006.

**Table 2:** Clinical features and management of heptic trauma over five chronological time periods in the decade studied

	2005–2006	2007–2008	2009–2010	2011–2012	2013–2014
*n*	373	487	780	1192	1536
Age					
Median (IQR)	31.6 (23.4–45.6)	33 (22.9–45.3)	30.7 (21.9–44.8)	34.4 (23.9–49.3)	35.3 (24.4–51.8)
Gender					
Male	277 (74.3%)	356 (73.1%)	571 (73.2%)	834 (70%)	1056 (68.8%)
Female	96 (25.7%)	131 (26.9%)	209 (26.8%)	358 (30%)	480 (31.3%)
Injury mechanism					
RTC	249 (66.8%)	295 (60.6%)	433 (55.5%)	673 (56.5%)	891 (58%)
Fall > 2 m	36 (9.7%)	49 (10.1%)	74 (9.5%)	141 (11.8%)	192 (12.5%)
Fall < 2 m	8 (2.1%)	13 (2.7%)	26 (3.3%)	62 (5.2%)	104 (6.8%)
Shooting/stabbing	63 (16.9%)	92 (18.9%)	182 (23.3%)	224 (18.8%)	235 (15.3%)
Blow(s)	9 (2.4%)	14 (2.9%)	34 (4.4%)	51 (4.3%)	67 (4.4%)
Other	8 (2.1%)	24 (4.9%)	31 (4%)	41 (3.4%)	47 (3.1%)
Charlson comorbidity index					
Not recorded	21 (6%)	44 (9.5%)	79 (10.3%)	141 (11.8%)	141 (9.2%)
None	120 (34.1%)	137 (29.7%)	201 (26.3%)	313 (26.3%)	488 (31.8%)
Minor: 1–5	89 (25.3%)	132 (28.6%)	223 (29.2%)	307 (25.8%)	373 (24.3%)
Moderate: 6–10	71 (20.2%)	103 (22.3%)	144 (18.8%)	237 (19.9%)	317 (20.6%)
Severe > 10	51 (14.5%)	45 (9.8%)	117 (15.3%)	194 (16.3%)	217 (14.1%)
Liver severity					
Median (IQR)	2 (2–4)	2 (2–3)	2 (2–3)	2 (2–3)	2 (2–3)
Liver isolated					
No	309 (82.8%)	383 (78.6%)	601 (77.1%)	955 (80.1%)	1217 (79.2%)
Yes	64 (17.2%)	104 (21.4%)	179 (22.9%)	237 (19.9%)	319 (20.8%)
GCS on arrival					
Median (IQR)	15 (8–15)	15 (13–15)	15 (13–15)	15 (13–15)	15 (13–15)
SBP on arrival					
Median (IQR)	125 (102–143)	120 (102–140)	122 (104–140)	122 (103–140)	123 (105–139)
Pulse on arrival					
Median (IQR)	96 (80–117)	95 (78–110)	93 (76–115)	93 (78–110)	90 (75–109)
Consultant at ED					
*n* (%)	172 (46.1%)	204 (41.9%)	430 (55.1%)	801 (67.2%)	1254 (81.6%)
TXA at ED					
*n* (%)	0 (0%)	0 (0%)	3 (0.4%)	196 (16.4%)	733 (47.7%)
CT					
*n* (%)	247 (66.2%)	363 (74.5%)	617 (79.1%)	997 (83.6%)	1357 (88.3%)
Time to CT (h)					
Median (IQR)	1.58 (0.97–2.5)	1.43 (0.85–3.12)	1.27 (0.65–2.9)	1 (0.52–2.45)	0.65 (0.35–1.62)
Surgery					
*n* (%)	212 (56.8%)	316 (64.9%)	470 (60.3%)	649 (54.4%)	809 (52.7%)
Liver surgery					
*n* (%)	9 (2.4%)	13 (2.7%)	39 (5%)	75 (6.3%)	105 (6.8%)
Admission to critical care					
*n* (%)	220 (59%)	299 (61.4%)	478 (61.3%)	721 (60.5%)	901 (58.7%)
LOS in critical care					
Median (IQR)	5 (2–11)	5 (2–11)	4 (2–10)	4 (2–10)	4 (1–9)
LOS					
Median (IQR)	11 (4–22)	11 (5–26)	9 (5–20)	9 (4–19)	9 (5–18)

**Table 3:** Patients undergoing liver specific surgery over the five time periods

	2005–2006	2007–2008	2009–2010	2011–2012	2013–2014
*n*	9	13	39	75	105
Age	35.2 (27.2–47.7)	33.9 (27.3–47.8)	29.5 (23.3–51.2)	34.1 (22.4–45.9)	31.9 (24.5–49.2)
Gender					
Male	7 (77.8%)	6 (46.2%)	31 (79.5%)	54 (72%)	71 (67.6%)
Grade of liver injury					
1	0 (0%)	5 (38.5%)	8 (20.5%)	16 (21.3%)	15 (14.3%)
2	4 (44.4%)	1 (7.7%)	12 (30.8%)	17 (22.7%)	37 (35.2%)
3	1 (11.1%)	2 (15.4%)	5 (12.8%)	23 (30.7%)	24 (22.9%)
4	4 (44.4%)	2 (15.4%)	9 (23.1%)	9 (12%)	20 (19%)
5	0 (0%)	3 (23.1%)	5 (12.8%)	8 (10.7%)	9 (8.6%)
9	0 (0%)	0 (0%)	0 (0%)	2 (2.7%)	0 (0%)
Charlson comorbidity index					
Not recorded	0 (0%)	2 (15.4%)	2 (5.3%)	5 (6.7%)	5 (4.8%)
None	3 (33.3%)	2 (15.4%)	7 (18.4%)	20 (26.7%)	25 (23.8%)
Minor: 1–5	1 (11.1%)	6 (46.2%)	13 (34.2%)	21 (28%)	36 (34.3%)
Moderate: 6–10	2 (22.2%)	3 (23.1%)	6 (15.8%)	18 (24%)	20 (19%)
Severe > 10	3 (33.3%)	0 (0%)	10 (26.3%)	11 (14.7%)	19 (18.1%)
ISS	48 (18–50)	41 (21–57)	34 (18–43)	29 (13–41)	29 (13–43)
SBP on arrival	104 (86–131)	95 (82–114)	99 (75–128)	106 (80–124)	111 (92–133)
Pulse on arrival	114 (111–128)	118 (100–148)	116 (88–130)	108 (90–130)	107 (80–122)
HPB unit	9 (100%)	6 (46.2%)	25 (64.1%)	42 (56%)	84 (80%)
Alive at 30 days	8 (88.9%)	9 (69.2%)	29 (74.4%)	57 (76%)	86 (81.9%)

**Table 4:** Logistic regression model for predictors of mortality for hepatic trauma in the year 2005–2014

	Coefficients		*p* value	Odds ratio (OR)	95% CI for OR	
					Lower	Upper
Model predicting survival						
16–44 (reference)						
45–54	−0.335		0.057	0.716	0.507	1.010
55–64	−0.741		0.001	0.476	0.314	0.724
65–75	−1.623		0.000	0.197	0.127	0.306
>75	−2.699		0.000	0.067	0.044	0.104
Male (reference)						
Female	0.135		0.284	1.144	0.894	1.465
ISS	−0.066		0.000	0.936	0.929	0.944
GCS 15 (reference)			0.000			
GCS = 3	−3.667		0.000	0.026	0.016	0.041
GCS 4–5	−2.138		0.000	0.118	0.046	0.301
GCS 6–8	−1.627		0.004	0.197	0.065	0.591
GCS 9–12	−2.174		0.000	0.114	0.038	0.342
GCS 13–14	−1.045		0.003	0.352	0.176	0.701
Intubated	−3.118		0.000	0.044	0.031	0.063
Not recorded	−0.947		0.000	0.388	0.275	0.548
2005–2009 (reference)			0.206			
2010–2012	0.093		0.517	1.097	0.829	1.453
2013–2014	0.259		0.084	1.296	0.966	1.738
CCI 0 (reference)			0.000			
CCI 1–5	0.232		0.136	1.261	0.929	1.712
CCI 6–10	0.664		0.000	1.943	1.361	2.773
CCI > 10	0.545		0.004	1.724	1.192	2.493
CCI not recorded	−1.090		0.000	0.336	0.236	0.479
Treated in HPB	1.259		0.000	3.521	2.745	4.516
Constant	4.550		0.000	94.661		

### Predictors of mortality

A logistic regression model for predictors of mortality for hepatic trauma in the year 2005–2014 is shown in Table 4. This includes hospitals known to have specialised hepato-pancreatico-biliary (HPB) units. Predictors of mortality after hepatic trauma were: increasing age and ISS, reduced Glasgow Coma Scale, haemodynamic compromise and Charlson comorbidity index (CCI) >10. Being treated in a unit with an on-site HPB service increased the odds of survival (odds ratio 3.5, 95% confidence intervals 2.7–4.5).

## Discussion

This study reports the frequency and extent of hepatic trauma in England, submitted to TARN, depicting the clinical features, management and outcomes of these injuries over a decade. Hepatic injury is mainly found in a young male population, is due to blunt trauma and predominantly managed conservatively, conforming with other large, contemporary, population-based studies in Europe [5], the Far East [6] and the USA [3, 11]. A predominance of penetrating trauma is found in single-centre South African studies, quoting rates of over 90%, with an increased need for surgical intervention [20, 21], compared to only 19% over-all in our series and 22% in a contemporary North American series [11]. This is attributed to the on-going predominance of firearm injuries in South Africa [20].

In each year of this study the frequency of liver injuries reported to TARN has increased. This may be due to the total increase in trauma volume in the UK as well as the accuracy of reporting and recording of trauma cases (TARN membership increased from 50 to 100% of all trauma receiving hospitals in England and Wales over the study period). The over-all mortality rate in this series of 16.4% compares favourably to the other UK (Scottish) based series reporting outcomes over the period 1992–2002 [5], which had a mortality rate of 42%. This high rate was largely attributed to the inclusion of patients who presented with catastrophic head injury, spinal cord transection, thoracic injuries, or massive haemorrhage who subsequently had liver injuries discovered at post mortem. The American College of Surgeons’ National Trauma Data Bank reported 35,510 hepatic injuries from 1994 to 2003. Over-all liver injury mortality remained relatively constant, averaging 16.8% [11]. In Taiwan, an over-all mortality rate of 8.3% was reported in 3196 cases of hepatic trauma admitted between 2007 and 2008 [6]. Mortality rates were significantly increased in the patients aged over 64 years; with head, chest, or other abdominal injuries; and with associated renal failure or liver cirrhosis [6]. Similarly, our study found that not only the severity of liver trauma but also the severity of concomitant extrahepatic injuries and existing comorbidities, as measured by the Charlson comorbidity index, had a major impact on survival.

Trends toward the non-operative management of blunt hepatic trauma have been widely documented [9, 10, 22, 23] with recent evidence showing that the integration of CT in early trauma-room management and a shift towards conservative management in hemodynamically stable patients results in improved survival in most [8, 10] but not all [12] series. Our series demonstrated an over-all 5.5% rate of liver specific operations compared to 16.7% in Taiwan [6] and 8.1% in the USA [11]. This emphasis on non-operative management has reduced the exposure of surgeons to operative intervention for liver trauma.

Our study has shown that being treated in a unit with an on-site HPB service increased the odds of survival. There is an improvement in risk adjusted mortality over time which disappears when the logistic regression model includes care in a HPB centre, therefore the observed improvement over time can be attributed to increased exposure to specialist HPB care. The mechanism whereby improved outcomes are associated with increasing HPB specialisation and centralisation in high volume centres may include: HPB surgeons having experience in complex haemostatic techniques that are not routinely used by others; training in vascular and transplant surgery; and detailed understanding of the complexities of liver anatomy. Other factors, such as recognising the need for experienced and early assistance and familiarity with managing post-operative complications (such as bile leaks) also likely contribute. Moreover, institutional factors, such as on-call interventional radiology and endoscopy, experienced theatre and nursing staff greatly contribute to the volume-outcome effect in hepatobiliary surgery [24]. One implication of our study is the need to improve training and exposure to hepatobiliary and pancreatic trauma surgery. Attachments to HPB units during general surgical training will facilitate this. Trauma courses, often using cadavers and simulation, are available and trainees should be encouraged to undertake these.

Chronological improvements in management found in this study included promptness of CT scanning and increased use of tranexamic acid. Our series reported the highest rate of CT scanning in 2013–2014 (88.9%). Recommendations on the use of CT for hemodynamically stable patients are well established according to Advanced Trauma Life Support (ATLS^®^) principles. The remaining 11% of patients may have warranted an immediate laparotomy due to haemodynamic instability or peritonitis, as recommended in other trauma practice management guidelines [25]. In the TARN registry detailed hepatic injury descriptors were more commonly obtained from ultrasound scans and operative or autopsy reports in the earlier years of the study, explaining the lower rates of CT scanning. Detailed information about the imaging modality used is often not available in other large series [3, 6, 11]. In a study spanning 25 years, Petrowsky et al. [8] examined changes in diagnostic modalities in hepatic trauma, finding that in the early study period (1986–1996), the majority of liver injuries were diagnosed by diagnostic peritoneal lavage followed by laparotomy. 72% were diagnosed by CT from 1997 to 2010, with this mode of scanning subsequently replacing ultrasound as the gold standard for diagnosis [8]. Use of tranexamic acid became routine after 2011, when the results of the CRASH-2 trial showed reduced mortality in trauma patients treated with early tranexamic acid [26].

The main strength of this study lies in the robust national data collection (using the electronic data collection and reporting system) and trained independent coders with stringent validation procedures. The series focussed on a time period where rapid changes were occurring to trauma service provision and HPB services in the UK. Liver trauma outcomes are favourable in the face of this evolving change. Further broad-based analyses comparing trauma outcomes prior to and after the formal accreditation of major trauma centres (MTCs) in 2010 and 2012 are also required to gain an appreciation of the changes that these have achieved. Thus far, significant improvements in the proportions of patients discharged with a good recovery (compared to disability) have been recorded [27] but overall improvements in mortality may take longer to demonstrate.

In summary, our series of over 4000 patients with hepatic trauma indicates that UK mortality rates are comparable to other large international series. Increased exposure to specialist hepatopancreatico-biliary teams in major trauma cases has improved outcomes for hepatic trauma in England and Wales over this decade.
